# Rac1 in human diseases: The therapeutic potential of targeting Rac1 signaling regulatory mechanisms

**DOI:** 10.1080/21541248.2016.1211398

**Published:** 2016-07-21

**Authors:** Hadir Marei, Angeliki Malliri

**Affiliations:** Cell Signaling Group, Cancer Research UK Manchester Institute, The University of Manchester, Manchester, UK

**Keywords:** cancer, cardiovascular diseases, Rac1, guanine nucleotide exchange factors (GEFs), GTPase activating proteins (GAPs), guanine nucleotide dissociation inhibitors (GDIs), inflammatory disorders, kidney diseases, neurodegenerative disorders, post-translational modifications, Rac1 targeting

## Abstract

Abnormal Rac1 signaling is linked to a number of debilitating human diseases, including cancer, cardiovascular diseases and neurodegenerative disorders. As such, Rac1 represents an attractive therapeutic target, yet the search for effective Rac1 inhibitors is still underway. Given the adverse effects associated with Rac1 signaling perturbation, cells have evolved several mechanisms to ensure the tight regulation of Rac1 signaling. Thus, characterizing these mechanisms can provide invaluable information regarding major cellular events that lead to aberrant Rac1 signaling. Importantly, this information can be utilized to further facilitate the development of effective pharmacological modulators that can restore normal Rac1 signaling. In this review, we focus on the pathological role of Rac1 signaling, highlighting the benefits and potential drawbacks of targeting Rac1 in a clinical setting. Additionally, we provide an overview of available compounds that target key Rac1 regulatory mechanisms and discuss future therapeutic avenues arising from our understanding of these mechanisms.

## Introduction

Ras-related C3 botulinum toxin substrate 1 (Rac1) is a member of the Rac family of guanosine triphosphate phosphohydrolases (GTPases), a subfamily of the Rho family of small GTPases, which are best known for their role in regulating the cytoskeleton and gene expression. Since its discovery, Rac1 has been implicated in various downstream cellular functions, including, but not limited to, cellular plasticity, migration and invasion, cellular adhesions, cell proliferation, apoptosis, reactive oxygen species (ROS) production and inflammatory responses, all of which are central to normal cell physiology.^1-3^ However, deregulation of Rac1 signaling can have detrimental effects since Rac1-driven cellular processes are also involved in a number of pathological conditions, including cancer,^4-6^ cardiovascular diseases,^7^ neurodegenerative disorders,^8^ pathological inflammatory responses,^9,10^ kidney disorders^11-16^ and infectious diseases.^17-19^ As such, Rac1 presents an attractive therapeutic target for combatting a number of human diseases. Yet, to effectively target Rac1 in a clinical setting, a proper understanding of its regulation is required.

Given its importance in normal cell physiology and the consequences associated with deregulation of Rac1 signaling, cells have evolved various mechanisms by which Rac1 signaling is restricted both spatially and temporally. Similarly to other small GTPases, Rac1 is a nucleotide-binding protein that associates with both guanosine diphosphate (GDP) and guanosine triphosphate (GTP), which leads to Rac1 inactivation or activation, respectively. Thus, Rac1 is mainly regulated via modulating its GDP-GTP binding, through the actions of guanine nucleotide exchange factors (GEFs), GTPase activating proteins (GAPs) and Rho guanine nucleotide dissociation inhibitors (RhoGDIs) Rac1.^1-3^ Additionally, Rac1 signaling is also modulated through post-translational modifications that dictate its activation status, abundance and localization.^20-36^ It has also become apparent that several factors influence Rac1 downstream signaling, via coupling active Rac1 to specific downstream effectors, thereby selectively activating certain Rac1-driven functions.^3,31,33,37-46^ Given the diverse modes involved in Rac1 regulation, studies focused on deciphering the underlying mechanisms implicated in governing Rac1 signaling promise to provide insight into novel therapeutic avenues for effective Rac1 targeting. In particular, uncovering additional Rac1 signaling regulatory cascades will help pinpoint key players in Rac1 signaling, thus expanding the repertoire of potential pharmacological targets that could be utilized to antagonize Rac1 signaling deregulation in human diseases.

In this review, we explore the benefits and potential drawbacks of targeting Rac1 in a clinical setting, via outlining the role of Rac1 in a number of human diseases. We also provide an overview of available compounds that target key Rac1 regulatory mechanisms and discuss future therapeutic avenues arising from both our current understanding of these mechanisms and the recent advancements in drug discovery screening methodologies.

## Rac1 signaling in human diseases

### Role of Rac1 in cancer

It has long been established that Rac1 drives tumor initiation, via serving as a Ras downstream effector, with *in vitro* data demonstrating the requirement of Rac1 for full oncogenic Ras transformation of NIH3T3 cells.^47^ *In vivo* studies have also highlighted the importance of Rac1 in both Kirsten rat sarcoma viral oncogene (K-Ras)-induced lung cancer^48^ and Harvey rat sarcoma viral oncogene (H-Ras)-induced skin cancer.^49^ Similarly to other Rho GTPases, Rac1 is also implicated in cell cycle progression, gene transcription and the release of pro-angiogenic factors and subsequent promotion of neovascularization, thereby promoting cancer initiation, progression and metastasis.^50-52^ In addition, Rac1 plays a critical role in mediating cell motility and invasion, 2 major steps in the metastatic cascade.^52-54^ For example, Rac1 drives the mesenchymal mode of cell migration, via stimulating the formation of actin-rich membrane extensions, such as lamellipodia, regulating the assembly of cell-extracellular matrix (ECM) focal adhesions as well as mediating myosin light chain (MLC) phosphorylation and cell contraction.^4,5,44,46,52,55,56^ Additionally, Rac1 facilitates cancer cell invasion via controlling the expression and release of matrix metalloproteinases (MMPs), which are required for ECM proteolytic degradation.^57-59^ Rac1 is also implicated in epithelial-mesenchymal transition (EMT) and mesenchymal-epithelial transition (MET), key events in the metastatic cascade of epithelial tumors, via mediating cellular plasticity and ECM modulation.^60-66^ More recently, an *in vivo* study also revealed that epidermis-specific activation of Rac1 in a transgenic mouse model of differentiated sebaceous adenomas, while not affecting tumor initiation, was associated with the formation of less differentiated tumors that resemble malignant sebaceous tumors, thereby implicating Rac1 in the malignant progression of sebaceous skin tumors.^67^

The importance of Rac1 in cancer is further demonstrated by the reported deregulation of Rac1 protein level and activity in a variety of tumors, which, in turn, facilitates tumor initiation, progression and metastasis.^6,52,68^ For example, Rac1 overexpression has been implicated in the initiation and progression of gastric, testicular and breast cancers.^69-71^ Overexpression of a splice variant of Rac1, designated Rac1b, has also been reported in a number of tumor types, including colorectal cancer, breast cancer and lung cancer.^71,72,73^ Unlike Rac1, Rac1b harbors an additional 57 nucleotides, leading to an in-frame insertion of 19 amino acids immediately following Rac1's switch II domain. Interestingly, Rac1b is predominantly present in the active GTP-bound form. This is attributed to a number of characteristics, including a high intrinsic guanine nucleotide exchange activity, granting Rac1b independence from GEFs, coupled with a reduced GTPase activity and impaired RhoGDI binding. Altogether, this enables Rac1b to maintain its activated state. However, given the location of the insertion and the role of switch I and II in mediating Rac1 association with downstream effectors, Rac1b displays impaired binding to a number of known Rac1 effectors, including p21 activated kinases (PAKs), leading to the activation of selective Rac1 downstream signaling cascades.^74-76^ Importantly, expression of Rac1b was shown to promote growth transformation in NIH3T3 cells.^76^ Additionally, depletion of Rac1b in colorectal cancer cells results in a significant reduction in cell viability, demonstrating the importance of Rac1b overexpression for colorectal cancer cell survival.^77^ More recently, *in vivo* data also implicated Rac1b in lung cancer initiation and progression, with expression of Rac1b in lung epithelial cells enhancing spontaneous tumor formation and promoting EMT.^78^ Additionally, similarly to Rac1, Rac1b is also required for K-Ras-induced lung cancer in transgenic mouse models.^73^ Given its role in cancer, together with its unique characteristics and limited downstream signaling cascades, Rac1b, thus, presents an attractive therapeutic target, with potentially limited off-target effects. Intriguingly, though, expression of Rac1b was shown to interfere with Rac1 activation and proper plasma membrane localization *in vitro*, while enhancing the activation of the closely related small GTPase Ras homolog gene family member A (RhoA).^79^ Although, the mechanism and functional significance of this Rac1b-mediated regulation of Rac1 and RhoA is yet to be fully elucidated, it would be important to determine whether alleviating this regulation via targeting Rac1b might confer drug resistance in cancer cells, due to increased Rac1 activation.

Until recently, overexpression of both Rac1 and its splice variant Rac1b, together with deregulation of Rac1 regulators was considered the major mode of Rac1 signaling perturbation in cancer.^6^ However, advancements in screening methodologies have also enabled the identification of a number of activating mutations in Rac1. Through exome sequencing of 147 melanomas, an activating Rac1 mutation was identified in 9.2 % of sun-exposed melanomas in which proline 29, located in the highly conserved switch I region, is replaced by serine (P29S).^80^ This mutation was also identified in a separate study aimed at mapping driver mutations in melanoma, further supporting a role for this this mutation in melanoma progression.^81^ Whole exome sequencing data from 74 tumor and normal sample pairs also identified Rac1 P29S as an activating mutation in head and neck squamous cell carcinoma.^82^ Moreover, following the sequencing of human cancer cell lines, Rac1 P29S was also detected in the breast cancer cell line MDA-MB-157. Additional Rac1 activating mutations were also uncovered, including the replacement of asparagine 92 with isoleucine (N92I) and cysteine 157 with tyrosine (C157Y).^83^ Interestingly, further analysis of the crystal structure of the Rac1 P29S mutant revealed that this mutation enhances Rac1 binding to its downstream effectors due to relieving the conformational restraints normally imposed by proline 29. This, in turn, leads to prolonged Rac1 downstream signaling, which promotes melanocyte proliferation and migration.^80^ It is unclear whether Rac1 N92I and Rac1 C157Y induce similar conformational changes as observed with Rac1 P29S, however all 3 mutations are associated with a rapid nucleotide exchange, thus favoring the GTP-bound state of Rac1. All 3 mutations were also found to be highly transforming in a number of cancer cell lines.^83^ Given their prevalence in a large number of cancers,^83^ together with evidence implicating mutations, such as Rac1 P29S in conferring resistance against B-Raf proto-oncogene, serine/threonine kinase (BRAF) inhibitors in melanoma,^84^ Rac1 activating mutants, similarly to Rac1b, represent attractive anti-cancer therapeutic targets. However, a better understanding of their downstream signaling cascades and their signaling overlap with wild type Rac1 would be important, in order to exploit their potential therapeutic benefit.

It is important to note that Rac1 also drives anti-tumorigenic effects. This is mainly attributed to its role in maintaining cadherin-mediated cell-cell contacts. For example, expression of constitutively active Rac1 was shown to antagonize Ras transformation of Madin-Darby canine kidney II (MDCKII) cells, by restoring epithelial morphology.^85^ Metastasis suppressor-1 (Mtss1) was also reported to regulate E-cadherin cell-cell adhesion stability through Rac1, with expression of Mtss1 resulting in reduced hepatocyte growth factor (HGF)-induced cell scattering through promoting stronger cell-cell contacts. It has, thus, been proposed that the observed loss of Mtss1 in a number of cancers contributes to increased metastasis through diminishing Rac1-mediated stabilization of cell-cell contacts.^86^ Consistently, expression of T-cell lymphoma invasion and metastasis-1 (Tiam1), a Rac-specific GEF, or constitutively active Rac1 in the renal cell carcinoma cell line ClearCa-28 inhibits cell migration, via enhancing E-cadherin-mediated adhesions. In addition to the role of Rac1 in regulating cell-cell contacts, Tiam1-Rac1 signaling was also shown to impede cellular invasion through the upregulation of tissue inhibitor of metalloproteinase-1 (TIMP-1) and -2 (TIMP-2), thereby counteracting MMP-induced ECM degradation.^87^ The protective role of Rac1 in cancer has also been demonstrated *in vivo*. For example, while far fewer skin tumors developed in carcinogen-treated Tiam1 knockout mice, proportionally they more often progressed to malignancy, thus indicating that Tiam1-Rac1 signaling inhibits cancer progression.^88^ Similarly, Tiam1 deficiency in adenomatous polyposis coli (APC) mutant multiple intestinal neoplasia (Min) mice reduced polyp growth while enhancing the migration and invasion of the intestinal tumors formed when compared to mice expressing Tiam1.^89^

Taken together, it is evident that deregulation of Rac1 signaling can drive tumor initiation, progression and metastasis, making it an attractive therapeutic target. However, given the contrasting roles of Rac1 in cancer, a thorough understanding of factors that influence Rac1 downstream specificity and biological output is needed prior to targeting Rac1 in a clinical setting.

### Role of Rac1 in cardiovascular diseases

In addition to cancer, deregulation of Rac1 signaling has been shown to drive cardiovascular diseases.^7^ For example, expression of constitutively active Rac1 in cardiomyocytes is associated with sarcomeric reorganization, increased cell size as well as the induction of atrial natriuretic factor (ANF) expression, all of which are characteristic of pathological cardiomyocyte hypertrophy, for example as a consequence of mechanical stress and ischemic injury.^90^ *In vivo* studies also highlight the role of aberrant Rac1 activation in cardiovascular diseases. This is evident from the prominent cardiomyopathy phenotype associated with transgenic mice expressing constitutively active Rac1.^91^ Constitutive activation of Rac1 *in vivo* has also been shown to enhance the spontaneous development of cardiac hypertrophy, with mice being more susceptible to ischemic injury with notable increases in myocardial infarcted areas.^92,93^

In addition to increased Rac1 activation, pathological cardiomyocyte hypertrophy is also associated with enhanced ROS production.^7^ Interestingly, Rac1 plays a crucial role in the activation of nicotinamide adenine dinucleotide phosphate (NADPH) oxidases.^94^ For example, Rac1 regulates the assembly of NADPH oxidase 2 (NOX2) via binding to p67^*phox*^, an important cytosolic regulatory subunit, and mediating its interaction with gp91^*phox*^, the membrane-associated component of the oxidase.^94-96^ NADPH oxidases constitute a family of multi-subunit enzymes that generate the ROS superoxide anion (O_2_^.−^). Deregulation of NADPH oxidases and ROS overproduction have been extensively linked to cardiovascular diseases.^97^ Thus, it is likely that aberrant Rac1 activation is responsible, at least partially, for the increased ROS production observed in cardiovascular diseases. Indeed, expression of dominant-negative Rac1 has been shown to impede ROS production in cardiomyocytes following mechanical stress-induced cardiac hypertrophy.^98^ Similarly, Rac1 inactivation abolishes angiotensin II-induced ROS generation in cardiomyocytes.^99^ Importantly, cardiomyocyte-specific Rac1 deletion in a transgenic mouse model was associated with reduced NADPH activation and myocardial oxidative stress, which correlated with decreased cardiac hypertrophy despite being subjected to angiotensin II-induced hypertensive stress.^100^ All together, this demonstrates that Rac1-mediated modulation of NADPH assembly and activation as well as ROS production plays a critical role in the progression of cardiovascular diseases. Thus, developing compounds that specifically target this cascade, while sparing other Rac1 functions provides an important therapeutic avenue.

NADPH oxidases have also been shown to contribute to atherosclerosis,^101,102^ thereby implicating Rac1 in disease progression. Inhibition of NADPH and the associated ROS production attenuates aortic atherosclerosis *in vivo*.^103^ It, thus, follows that Rac1 inhibition or depletion would also mimic this phenotype. Rac1 also regulates a number of cellular processes that are associated with advanced atherosclerosis. For example, expression of dominant-negative Rac1 abolishes the migration of aortic smooth muscle cells (SMCs),^104^ which are known to accumulate in the inner layers of arteries during atherosclerosis.^105^ Rac1 signaling has also been shown to mediate SMCs, migration and accumulation in arterial walls in response to tumor necrosis factor-α (TNF-α) stimulation.^106^ Additionally, Rac1 regulates endothelial permeability, a key determinant of lipoproteins' entry in vascular walls. Intriguingly, although inactivation or depletion of Rac1 has been shown to enhance endothelial permeability,^107,108^ expression of Rac1 and the subsequent activation of its downstream effector PAK in endothelial cells enhances junction permeability through regulating adherens junctions as well as promoting cell contractility through MLC phosphorylation.^109^ Indeed, displacement of active PAK from endothelial cell-cell contacts or inhibition of its activity were shown to antagonize the role of vascular endothelial growth factor (VEGF), basic fibroblast growth factor (bFGF), histamine, TNF-α and thrombin in stimulating endothelial permeability.^109^ Consistently, inhibition of PAK *in vivo* reduces endothelial permeability in atherosclerosis-prone regions.^110^ This indicates that increased activation of Rac1-PAK signaling can stimulate atherosclerosis, through enhancing the deposition of lipoproteins on vasculature walls. It also highlights the importance of downstream signaling specificity in determining the biological output downstream of Rac1, which is of particular relevance for the effective targeting of aberrant Rac1 signaling.

Rac1 also plays a key role in mediating the inflammatory response associated with cardiovascular diseases. For example, the movement of leukocytes from blood vessels into arterial walls marks a key step in the inflammatory response associated with atherosclerosis.^111^ Thus, through driving leukocyte trans-endothelial migration and accumulation in arterial walls, Rac1 contributes to inflammation in response to vascular injury.^112^ Together with regulating NADPH assembly and activation, the role of Rac1 in inflammation also implicates it in the formation of aortic aneurysms^113,114^ and the initiation and progression of chronic heart failure.^115^

Altogether, both *in vitro* and *in vivo* studies demonstrate the role of Rac1 in cardiovascular diseases. Through modulating actin cytoskeleton rearrangements, gene expression, cell adhesions and migration, Rac1 contributes to disease progression via affecting key processes, such as endothelial permeability, ROS production and the migration of SMCs and leukocytes. Thus, selective inhibition of these Rac1-driven processes might prove particularly useful for attenuating cardiovascular disease progression.

### Role of Rac1 in neurodegenerative diseases

Rac1 signaling plays an essential role in neuronal development, outgrowth, migration and plasticity. As such, deregulation of Rac1 signaling is also implicated in neurodegenerative diseases.^8^ Through regulating neuronal survival, it is apparent that Rac1 has a protective function against apoptosis-mediated neurodegeneration.^116-119^ In familial amyotrophic lateral sclerosis (ALS), a neuromuscular disorder characterized by the loss of motor neurons, cortex and upper and lower spinal cord, mutations of Cu, Zn superoxide dismutase 1 (SOD1) have been shown to contribute to the clinical manifestations of ALS. Interestingly, expression of constitutively active Rac1 attenuates neuronal death induced by SOD1 mutants.^120^ Mutations in alsin, a Rac1 GEF, have also been reported in familial ALS as well as other motor neuron diseases, such as primary lateral sclerosis and infantile-onset ascending hereditary spastic paralysis.^121^ Under physiological conditions, alsin serves as a Rac1 GEF that mediates Rac1-driven neurite outgrowth.^122^ Intriguingly, alsin mutants associated with motor neuron diseases are rapidly degraded when expressed in human cells.^123^ This suggests that the loss-of-function of alsin, and by association failure to activate Rac1, might play a key role in the propagation of neurodegeneration. Indeed, knockdown of alsin in murine spinal motor neurons suppresses neuronal outgrowth and induces cell death, effects that are abrogated by the expression of constitutively active Rac1.^117^ Additionally, alsin has been shown to antagonize the effects of SOD1 mutants on motor neuronal death in a Rac1-dependent manner,^116^ further highlighting the protective role of Rac1 in neurodegenerative diseases.

The protective function of Rac1 also extends to Parkinson's disease, a neurodegenerative locomotive, cognitive and behavioral disorder that is caused by the degeneration of the nigrostriatal dopaminergic neurons of the midbrain. Mutations in leucine-rich repeat kinase 2 (LRRK2) are considered the most common genetic cause of familial Parkinson's disease.^124^ Interestingly, expression of Rac1 rescues neurite retraction and neuronal cell death induced by LRRK2 mutants.^125^ This suggests that, similar to ALS, Parkinson's disease progression might require diminished Rac1 activity.

Intriguingly, despite the apparent protective role of Rac1 in neurodegenerative diseases, SOD1 mutations in microglia have been shown to enhance Rac1 activity. This, in turn, promotes Rac1-driven activation of NADPH oxidase and ROS overproduction,^126,127^ which are known to contribute to the motor neuron degeneration attributed to SOD1 mutations.^128^ Similarly, abnormal ROS production has also been linked to the progression of Huntington's disease, a neurodegenerative disorder with debilitating clinical manifestations, including involuntary body movement, cognitive impairment and eventually death. This is clearly demonstrated by the enhanced survival associated with pharmacological inhibition and genetic deletion of NADPH oxidase in Huntington's disease mouse models.^129^ In addition to its role in ROS production, Rac1 and its downstream effector PAK have been shown to interact with mutant huntingtin in a yeast 2-hybrid screen.^130^ Mutant huntingtin forms toxic protein aggregates that correlate with disease onset. Thus by directly binding to mutant huntingtin, Rac1 might directly contribute to the development of Huntington's disease.

Rac1 signaling is also implicated in Alzheimer's disease, a neurodegenerative disorder characterized by neuronal loss in the hippocampus and cerebral cortex. A hallmark of Alzheimer's disease is the aberrant accumulation of extracellular amyloid-β plaques, which correlates with both disease onset and synaptic dysfunction observed during disease progression.^131^ Interestingly, Rac1 has been shown to regulate the transcription and expression of the amyloid precursor protein (APP), the proteolytic cleavage precursor of amyloid-β.^132^ Additionally, inhibition of Rac1 was shown to reduce γ-secretase-dependent APP cleavage.^133^ Thus, via enhancing APP production and cleavage, Rac1, likely, plays an important role in the formation of the amyloid-β plaques observed in Alzheimer's disease.

As highlighted, similar to cancer, Rac1 is associated with both protective and promoting effects in neurodegenerative diseases. Thus, to exploit the therapeutic potential of targeting Rac1 it is important to design pharmacological tools that selectively inhibit Rac1 signaling cascades involved in disease progression while sparing its protective functions. However, this requires a detailed understanding of the cellular contexts in which Rac1 is to be targeted.

### Role of Rac1 in other human diseases

Rac1-driven signaling cascades highlighted above are also implicated in other human diseases. This is clearly demonstrated by the role of Rac1 in arthritis. Arthritis refers to a group of diseases that affect the joints, with more than 100 types identified to date. The underlying causes for arthritis can vary depending on the form of arthritis. For example, osteoarthritis, the most common form, affects joints due to the ware and tare of the protective cartilage and the underlying bones as a result of aging, injury or infection.^134^ Central to osteoarthritis is the accumulation of matrix fragments, such as fibronectin fragments, which induce the production of MMPs by chondrocytes. This, in turn, leads to the degradation of the of articular cartilage matrix.^135^ Interestingly, Rac1 was shown to mediate fibronectin fragment-induced MMP-13 production. Consistently, active Rac1 was detected in osteoarthritis cartilages.^136^ Rac1 was also shown to promote expression of ADAM metallopeptidase with thrombospondin type 1 motif 5 (ADAMTS-5),^137^ an enzyme responsible for cleaving aggrecan, a major cartilage proteoglycan. Additionally, Rac1 also stimulates the production of chondrocyte hypertrophy-related factors, such as type X collagen (COLX) and runt-related transcription factor 2 (Runx-2).^137^ Together, this indicates that Rac1 activation contributes to the development of osteoarthritis via facilitating cartilage matrix destruction. More recently, it was shown that downregulation of the Rac1 GAP, inositol polyphosphate 5-phosphatase OCRL-1 (OCRL1), in cartilages is responsible for the aberrant activation of Rac1 in osteoarthritis. Importantly, restoring physiological active Rac1 levels by re-expressing OCRL1 was associated with reduced chondrocyte hypertrophy *in vitro* and protected against cartilage degeneration *in vivo*.^138^ This clearly highlights the therapeutic potential of targeting Rac1 to protect against osteoarthritis. Indeed, recent *in vivo* evidence demonstrates that inhibition of Rac1 can help delay osteoarthritis development.^137^

In addition to promoting chondrocyte hypertrophy and mineralization, Rac1 is also implicated in the development of inflammatory arthritis. This is particularly evident in rheumatoid arthritis, a debilitating autoimmune disorder that is characterized by cartilage loss due to increased inflammation and catabolism of the joint lining. In rheumatoid arthritis, the immune system starts attacking the synovial membrane lining the joints. As such, rheumatoid arthritis is characterized by increased T-cell activation and autoantibody production. Together, this induces a localized inflammatory response that leads to the swelling of the synovial membrane, causing further damage and pain. Additionally, surrounding synovial cells, such as fibroblast-like synoviocytes, also display tumor-like properties.^139^ Given the prominent role of Rac1 in cancer, it is not surprising that Rac1 contributes to the proliferative and invasive properties of fibroblast-like synoviocytes isolated from rheumatoid arthritis patients. Importantly, both depletion of Rac1 and chemical inhibition was shown to antagonize the aggressive tumor-like properties of fibroblast-like synoviocytes.^140^ Targeting Rac1 using an inhibitory peptide was also shown to reduce paw swelling in early arthritis and to a lesser extent in chronic arthritis in a collagen-induced arthritis murine model. Additionally, while Rac1 inhibition did not protect against joint destruction in this model, it was associated with reduced levels of anti-collagen type II antibodies. *Ex vivo* analysis also demonstrated that Rac1 inhibition suppresses T-cell activation. Together, this suggests that targeting Rac1 could be of clinical relevance in autoimmune disorders, such as rheumatoid arthritis.^141^

In addition to inflammatory arthritis, Rac1 also contributes to the inflammation associated with kidney disorders.^15^ For example, depletion of Rac1 in macrophages was shown to suppress lipopolysaccharide (LPS)-inflammation-mediated kidney injury.^16^ Interestingly, Rac1 is hyperactivated in several chronic kidney disease models and is linked to the increased activation and nuclear translocation of the mineralocorticoid receptor, ^12-14^ a member of the steroid receptor family that plays an essential role in the progression of kidney diseases.^11^ Importantly, inhibition of Rac1 was shown to have renoprotective functions by counteracting the hyperactivation of the mineralocorticoid receptor.^12-14^ Thus, inhibition of Rac1 signaling also constitutes an effective treatment option for attenuating the progression of several kidney disorders.

Consistent with the role of Rac1 in mediating inflammatory responses, deregulation of Rac1 signaling is also implicated in inflammatory disorders. Increased expression of Rac1, induced by single nucleotide polymorphisms, has been shown to play a role in the pathogenesis of chronic inflammatory bowel diseases, including ulcerative colitis and Crohn's disease.^9^ In fact, thiopurines, a class of drugs currently utilized for the management of chronic inflammatory bowel diseases, have been shown to suppress Rac1 activity.^142,143^ Thus, inhibiting Rac1 activation and the associated inflammatory and immune responses might help alleviate clinical symptoms associated with inflammatory disorders.

Aberrant regulation of Rac1 signaling is also a key feature in a number of infectious diseases. Interestingly, a number of pathogens hijack Rac1 signaling in order to promote pathogenicity. For example, activation of Rac1 at early stages of *Salmonella typhimurium* bacterial infection has been shown to promote bacterial entry into host cells via mediating the necessary actin cytoskeletal rearrangements required for bacterial internalization.^17-19^ Similarly, activation of Rac1 has been implicated in the internalization of human immunodeficiency virus (HIV) and vaccinia virus. Additionally, Rac1 regulates the vesicular trafficking of viral particles of adenoviruses, African swine fever virus as well as Ebola virus.^144^ However, demonstrating the complexity of Rac1 signaling in infectious diseases, Rac1 is also implicated in the innate immune response.^145,146^ Therefore, targeting Rac1 to combat infection requires a detailed understanding of its regulation and downstream effector interactions in order to synthesize effective drugs that spare its protective functions.

## Targeting Rac1 signaling regulatory mechanisms provides multiple therapeutic avenues

**Figure 1.**
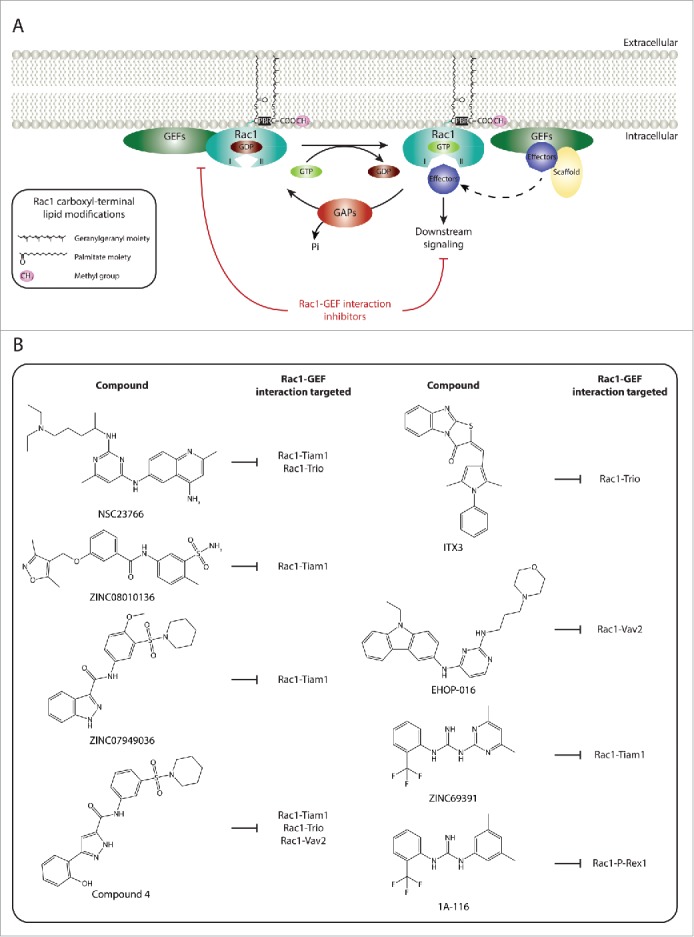
Targeting Rac1 activation and downstream signaling via blocking Rac1-GEF interactions. (A) Similarly to other Rho GTPases, Rac1 cycles between an inactive guanosine diphosphate (GDP)-bound state and an active guanosine triphosphate (GTP)-bound state. This cycle is regulated, in part, by guanine nucleotide exchange factors (GEFs) and GTPase activating proteins (GAPs). Following Rac1 prenylation, in which a geranylgeranyl moiety is covalently attached to cysteine 189, carboxyl-terminal methylation and the addition of a palmitate moiety on cysteine 178, GDP-bound Rac1 associates with the plasma membrane. The Polybasic region (PBR) of Rac1 has also been implicated in targeting Rac1 to the plasma membrane. This, in turn, allows GEFs present at the plasma membrane to bind to Rac1 and facilitate the exchange of GDP for GTP, thereby activating Rac1. As a result of GTP binding, Rac1 undergoes a conformational change in its switch I and switch II regions (depicted as I and II, respectively) that promotes binding with downstream effectors, thus translating upstream signals into downstream responses. In addition to Rac1 activation, GEFs can also serve as scaffolding proteins, via indirectly or directly associating to Rac1 effectors, thereby enriching specific Rac1-effector complexes and dictating Rac1 downstream signaling cascades. It is unclear, however, whether plasma membrane localization is essential for the GEF scaffolding function. In contrast, GAPs serve as Rac1 inhibitors, through enhancing the intrinsic GTPase activity of Rac1 and promoting the hydrolysis of bound GTP. (B) Given the importance of GEFs in activating Rac1 and mediating Rac1 downstream signaling, a number of Rac1 specific inhibitors have been developed that target Rac1 activation via blocking Rac1-GEF interactions. Examples of chemical structures and selectivity toward specific Rac1-GEF interactions are outlined.

**Figure 2.**
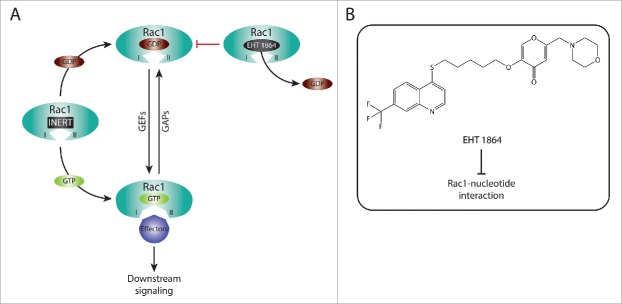
Targeting Rac1 activation and downstream signaling via blocking Rac1-nucleotide interactions. (A) Rac1 is a nucleotide-binding protein that associates with both guanosine diphosphate (GDP) and guanosine triphosphate (GTP). The dissociation of GDP is facilitated by guanine nucleotide exchange factors (GEFs), resulting in Rac1 GTP loading and activation. In contrast, GTPase activating proteins (GAPs) promote the hydrolysis of GTP, thus inactivating Rac1. Following GTP binding, Rac1 undergoes conformational changes in the switch I and switch II regions (depicted as I and II, respectively) that expose the effector binding domain, allowing Rac1 to bind to downstream effectors, thereby mediating downstream signaling. EHT 1864 is a selective Rac1 inhibitor that binds with high affinity to Rac1. This, in turn, promotes the dissociation of nucleotides bound to Rac1, placing Rac1 in an inert and inactive state that is unable to enter the activation GDP-GTP cycle or bind to downstream effectors. (B) The Chemical structure of EHT 1864 and its reported mode of action are outlined.

**Figure 3.**
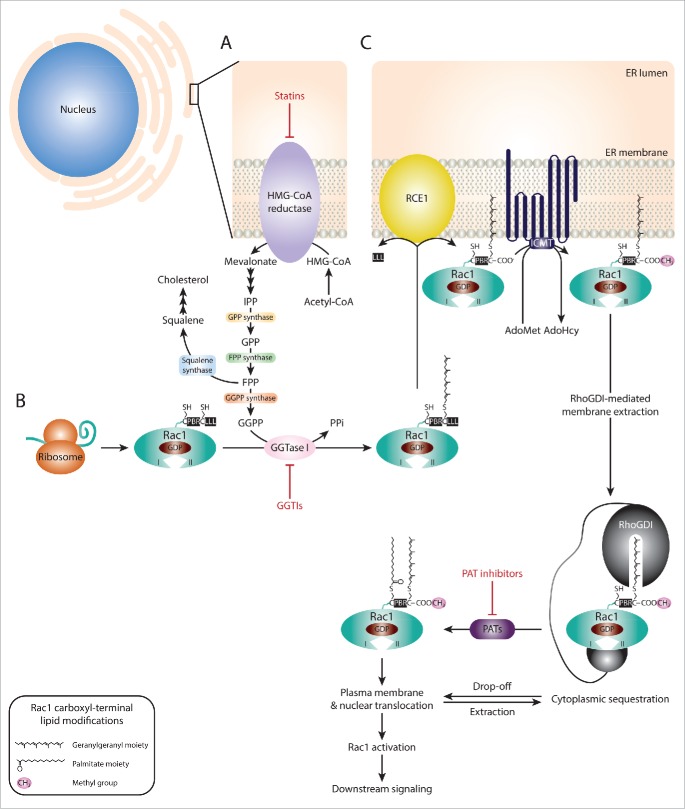
Targeting Rac1 activation and downstream signaling via blocking Rac1 lipid modifications. (A) The isoprenoid pathway is a multi-step chemical cascade, initiated by the 3-hydroxy-2-methylglutaryl-coenzyme A reductase (HMG-CoA reductase). HMG-CoA resides in the endoplasmic reticulum (ER) and represents a rate-limiting step in the conversion of acetyl-CoA into mevalonate, which is converted into isopentenyl diphosphate (IPP), the precursor of geranyl diphosphate (GPP) and farnesyl diphosphate (FPP). Via the action of squalene synthase, FPP can be converted into squalene, the committed precursor for cholesterol production. Alternatively, FPP can be converted into geranylgeranyl diphosphate (GGPP) by GGPP synthase. Both FPP and GGPP can serve as lipid backbones for protein prenylation, which involves the addition of a farnesyl or geranylgeranyl moiety to the cysteine residue within the CAAX motif (where C represents the cysteine, AA represent 2 aliphatic amino acids and X is the terminal amino acid) by farnesyltransferases (FTases) or geranylgeranyltransferases (GGTases), respectively. (B) Following Rac1 protein expression, the CAAX motif (CLLL) located at the carboxyl-terminus is recognized by GGTase type I (GGTase I). This results in the covalent attachment of a geranylgeranyl moiety to cysteine 189. (C) Rac1 prenylation promotes Rac1 translocation and association with the ER membrane where the RAS-converting CAAX endopeptidase (RCE1) and the isoprenylcysteine carboxyl methyltransferase (ICMT) mediate Rac1 post-prenylation modifications on the cytosolic surface of the ER. First, RCE1 cleaves the 3 amino acids (LLL) following the prenylated cysteine residue. This exposes the carboxyl-group of the cysteine amino acid (COO^−^), thus allowing the transfer of a methyl group (CH_3_) from S-adenosyl methionine (AdoMet) to Rac1 by ICMT, releasing S-adenosyl- _L_ homocysteine (AdoHcy) as a by-product. The fully processed prenylated Rac1 protein can then be trafficked to the plasma membrane. Rho guanine nucleotide dissociation inhibitors (RhoGDIs) have been implicated in mediating Rac1 extraction from the ER membrane, with the RhoGDI-Rac1 association being tightly regulated and reversible, thus allowing cycling between Rac1 membrane/nuclear translocation and cytoplasmic sequestration. Rac1 prenylation and processing also primes Rac1 for palmitoylation, in which a 16-carbon fatty acid palmitate is attached to cysteine 178 by palmitoyl acyltransferases (PATs). Rac1 palmitoylation also depends on the polybasic region (PBR) within the carboxyl-terminus of Rac1. It is unclear whether palmitoylation of Rac1 occurs in the cytoplasm or on the plasma membrane. It is also yet to be determined whether palmitoylation preferentially occurs on GDP-bound or GTP-bound Rac1, for simplicity the latter was not depicted in the figure. Rac1 palmitoylation enhances Rac1 stability and mediates Rac1 localization to the plasma membrane, particularly to lipid-ordered subdomains, as well as nuclear translocation. Statins, GGTase I inhibitors (GGTIs) and PAT inhibitors have been shown to target HMG-CoA reductase, GGTase I and PAT enzymes, respectively. As a result, all 3 classes of compounds have been shown to disrupt Rac1 localization, activation and downstream signaling.

**Figure 4.**
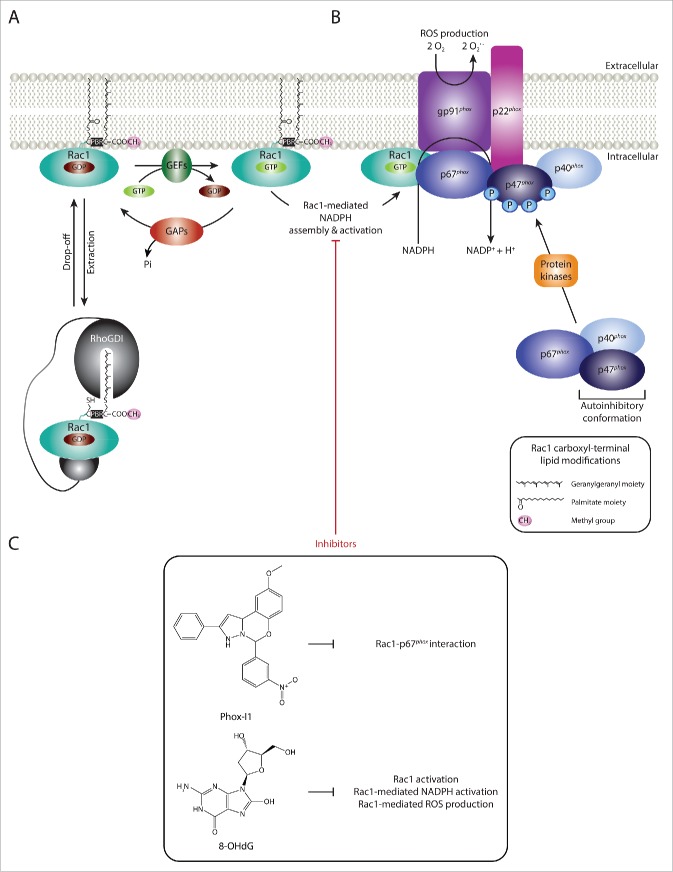
Targeting Rac1-mediated assembly and activation of NADPH oxidases and ROS production. (A) Guanine nucleotide exchange factors (GEFs), GTPase activating proteins (GAPs) and Rho guanine nucleotide dissociation inhibitors (Rho GDIs) regulate Rac1 cycling from an inactive guanosine diphosphate (GDP)-bound state to an active guanosine triphosphate (GTP)-bound state. RhoGDIs also play a role in Rac1 cytoplasmic sequestration. (B) Nicotinamide adenine dinucleotide phosphate (NADPH) oxidases are multimeric protein complexes that transfer electrons from intracellular NADPH to extracellular molecular oxygen, generating superoxide anions (O_2_^.−^), a reactive oxygen species (ROS), in the process. Rac1 has been shown to regulate the assembly and activation of NADPH oxidases. For simplicity only one NADPH oxidase isoform, NADPH oxidase 2 (NOX2), is depicted. Activation of the complex involves the assembly of the cytosolic regulatory proteins (p67^*phox*^, p40^*phox*^ and p47^*phox*^) with the membrane-associated components (the catalytic subunit gp91^*phox*^ and p22^*phox*^). This is mediated through the phosphorylation of the autoinhibitory region of p47^*phox*^, thereby releasing the autoinhibitory conformation and promoting p47^*phox*^-p22^*phox*^ interaction. Additionally, activated Rac1 has been shown to directly bind to the p67^*phox*^ subunit, thereby further facilitating complex assembly. Although not depicted in the figure, lipid metabolism within the plasma membrane also plays an important role in providing the anchoring sites for p40^*phox*^ and p47^*phox*^. (C) Given the role of Rac1-mediated NADPH assembly and activation and ROS production in the progression of a number of human diseases, inhibitors that specifically target this Rac1 downstream signaling cascade have been identified. Phox-I1 represents a Phox-I class inhibitor that functions via blocking the interaction between Rac1 and p67^*phox*^, thereby inhibiting Rac1-mediated complex assembly. Another example includes 8-hydroxy-2-deoxyguanosine (8-OHdG), which also inhibits Rac1-medaited NADPH activation and ROS production. The chemical structures and mode of action of both compounds are outlined.

As outlined above, deregulation of Rac1 signaling is characteristic of a number of human diseases, highlighting the therapeutic potential of targeting Rac1. However, given the overlap between its physiological and pathological functions as well as its often contrasting effects in cellular processes, targeting Rac1 in a clinical setting might have undesirable effects on disease progression. Understanding how Rac1 signaling is regulated can, therefore, provide additional insights to aid the development of pharmacological tools for targeting specific Rac1 downstream functions. Indeed, a number of compounds have been developed to date that suppress Rac1 signaling via targeting the various steps involved in its regulation (Figs. 1–4). Here we provide an overview of available compounds that target key Rac1 regulatory mechanisms and discuss future therapeutic avenues arising from our understanding of these mechanisms.

### Targeting Rac1 activation

Rac1 acts as a molecular switch cycling between an inactive GDP-bound state and an active GTP-bound state. Binding of GTP to Rac1 and its subsequent activation induces a conformational change that promotes binding to downstream effectors.^147^ As mentioned above, regulation of this GDP-GTP activation cycle is mediated mainly by 3 groups of proteins: GEFs, GAPs and RhoGDIs, with GEFs activating Rac1 while GAPs and RhoGDIs inhibiting Rac1 signaling.^1-3^

The role of GEFs in Rac1 signaling is particularly important. Through interacting with Rac1, GEFs facilitate the exchange of GDP for GTP by promoting GDP dissociation.^148^ In principle, GTP loading primes Rac1 for binding to all downstream effectors. However, accumulating evidence supports a role for GEFs, not only in activating Rac1, but also in dictating its downstream signaling, through influencing Rac1 effector specificity.^38-41,43-46^ This is clearly demonstrated by the reported role of Tiam1 and Ras protein-specific guanine nucleotide-releasing factor 1 (Ras-GRF1) as scaffolding proteins. Through binding to the scaffolding protein mitogen-activated protein kinase 8 interacting protein 2 (IB2/JIP2) and facilitating its interaction with specific components of the mitogen-activated protein kinases (MAPK) cascade, including the Rac1 effector mixed lineage kinase 3 (MLK3), mitogen-activated protein kinase kinase 3 (MKK3) and mitogen-activated protein kinase 14 (p38), both GEFs couple activated Rac1 to the p38 signaling cascade over the Jun N-terminal kinase (JNK) signaling cascade.^39^ In addition to IB2/JIP2, Tiam1 binding to spinophilin, another scaffolding protein, is also implicated in modulating Rac1 downstream signaling specificity. In particular, Tiam1-spinophilin complex formation was shown to increase Tiam1-Rac1-mediated p70 S6 kinase activation, while suppressing Tiam1-Rac1-mediated PAK activation.^40^ The observed selective activation of p70 S6 kinase following Tiam1-spinophilin binding is likely a consequence of increased Rac1 association with p70 S6, which was previously reported to enhance p70 S6 activation.^149^ Additionally, GEFs can also dictate Rac1 signaling, through directly associating with its downstream effectors. For example, Tiam1 was shown to bind directly to the Rac1 effector insulin receptor tyrosine kinase substrate p53 (IRSp53), thus enhancing its interaction with activated Rac1 and the WASP-family verprolin-homologous protein 2 (WAVE2).^41^ IRSp53 has previously been reported to provide a link between Rac1 and WAVE2 to mediate Rac1-driven actin polymerization and lamellipodia formation.^56,150^ Interestingly, Tiam1 expression also stimulates the localization of IRSp53 at Rac1-mediated lamellipodia, indicating that Tiam1 drives actin polymerization and lamellipodia formation through coupling Rac1 to IRSp53 and WAVE2.^41^ Examples of other GEFs that bind to Rac1 downstream effectors also include members of the PAK-interacting exchange factor (PIX) family of GEFs, which through their Src-homology 3 (SH3) domain, bind to PAKs.^38^ More recently, a similar scaffolding role was also reported for the Rac GEF phosphatidylinositol-3, 4, 5-trisphosphate-dependent Rac exchange factor 1 (P-Rex1). Through directly binding to protein flightess-1 homolog (FLII), an actin remodeling protein, P-Rex1 was shown to enhance Rac1-FLII association concomitant with increased FLII-dependent P-Rex1-driven MLC phosphorylation, cell contraction and ECM remodeling, thereby stimulating Rac1-driven cell migration. More importantly, expression of Tiam1, under the same cellular conditions, was not associated with increased Rac1-FLII interaction, indicating a GEF-specific modulation of Rac1-effector binding.^44^ Indeed, comparative quantitative proteomic analysis of the Rac1 interactome revealed that Tiam1 and P-Rex1 stimulate Rac1 association with GEF-specific effector pools that could account for the contrasting roles of these 2 GEFs in regulating Rac1-driven cell migration.^45,46^ Thus, all together this highlights the importance of the GEF scaffolding function in mediating specific Rac1-driven signaling cascades in response to different upstream cues.

#### Targeting Rac1-GEF interactions

Given the importance of GEFs in orchestrating Rac1 signaling and the potential therapeutic benefit of inhibiting selective Rac1 functions, disrupting Rac1 interactions with specific GEFs represents an attractive therapeutic avenue. Indeed, several Rac1 inhibitors identified have been shown to target specific Rac1-GEF associations (Fig. 1).

The search for selective inhibitors that target specific Rac1-GEF interactions was greatly facilitated by solving the crystal structure of Rac1 in complex with the Dbl homology (DH) and pleckstrin homology (PH) domains of Tiam1, which provided important details of the mechanism and specific sites involved in Rac1-GEF interactions.^151^ This information paved the way for structure-based virtual screening that led to the discovery of NSC23766, the first selective Rac1 inhibitor. Functional characterization of this compound revealed that it inhibits Rac1 activation via blocking the surface groove of Rac1, responsible for mediating GEF association, particularly between Rac1 and Tiam1 as well as between Rac1 and triple functional domain protein (Trio). *In vitro* validation assays using this compound demonstrated the specificity of NSC23766 in inhibiting Tiam1- and Trio-mediated cell growth and transformation while not affecting signaling cascades driven by the Rac GEF Vav or the activation of the closely related small GTPases, RhoA and cell division control protein 42 homolog (Cdc42).^152^ Interestingly, NSC23766-mediated Rac1 inhibition has been shown to inhibit Rac1-driven pro-tumorigenic effects in a number of cancer models.^153^ The protective effects of NSC23766 also extend to other disease models, including neurodegenerative and kidney disorders as well as arthritis.^8,12,137,140^ More recently, *in vivo* experiments also indicated that NSC23766-mediated inhibition of Rac1 might have anti-viral properties.^154^

Despite the promising results associated with NSC23766, this compound lacks the efficacy required for clinical purposes. This instigated a number of virtual screening strategies in search for more potent Rac1 inhibitors. Indeed, a pharmacophore virtual screening approach led to the identification of 5 compounds that selectively inhibit Rac1 GTP loading while not affecting RhoA and Cdc42 activation, 2 of which, ZINC08010136 and ZINC07949036, function through inhibiting Rac1-Tiam1 binding with potencies greater than NSC23766.^155^ The chemical structures of these 2 compounds were then used to virtually screen commercially available N-(sulfamoylaryl)arylamides, which led to the identification of 5 additional compounds that displayed yet even more potent and selective inhibition of Rac1 with IC_50_ values ranging from 5.3 to 24.2 μM as opposed to 50 μM observed with NSC23766. In particular, one of these compounds, referred to as compound 4, was shown to inhibit GEF-mediated Rac1 GTP loading via interfering with Rac1 binding to Tiam1, Trio and Vav2, thereby suppressing cell adhesion and Rac1-mediated cellular events.^156^ Additionally, preliminary *in vitro* analysis demonstrated that this compound suppresses platelet-derived growth factor-BB (PDGF-BB)-mediated Rac1-driven lamellipodia formation as well as SMCs' migration.^7^ These results may have important implications on the pathological role of Rac1 in cardiovascular diseases, although *in vivo* studies are still required to determine the efficacy of this compound in preclinical disease models.

ITX3 represents another selective Rac1 inhibitor that interferes with Rac1-Trio binding. While treatment of cells with ITX3 results in the selective inhibition of Trio N-terminal RhoGEF domain (TrioN)-dependent cell structures, the IC_50_ of ITX3 is 100 μM.^157^ As a result of its low efficacy, ITX3 is not ideal for clinical purposes. More recently, however, further optimization of the chemical structure of NSC23766 has led to the identification of more potent Rac1 inhibitors, such as EHop-016, which represents a highly potent Rac1 inhibitor with an IC_50_ of 1.1 μM. Similar to NSC23766, EHop-016 functions through interfering with Rac1-GEF binding. However, unlike NSC23766, EHop-016 does not affect Rac1-Tiam1 association. Instead EHop-016 blocks Rac1-Vav2 binding and has been shown to suppress Rac1-driven directed cell migration of metastatic cancer cells.^158^ This highlights the potential importance of EHop-016 and similar compounds in combating Rac1-mediated cancer metastasis, a major cause of death in cancer patients. Interestingly, EHop-016 has also been shown to decrease Rac1-mediated activation of PAK1. As highlighted earlier, PAKs play a major role in cardiovascular diseases via increasing endothelial membrane permeability, with inhibition of PAK suppressing endothelial permeability.^109,110^ Thus, EHop-016, through suppressing Rac1-mediated activation of PAK1, might have beneficial effects not only in cancer but also in other human diseases, including cardiovascular diseases. A drawback of this compound, however, is that it also targets Cdc42. This calls for additional preclinical studies in order to pinpoint potential side effects arising from its promiscuity.

Recently, yet another virtual screen was reported in which more than 200, 000 compounds from the ZINC database were screened to identify compounds that can interfere with Rac1-GEF binding.^159,160^ This led to the identification of a novel Rac1 inhibitor, ZINC69391, which was associated with high docking scores for the Rac1-GEF interface, suggesting a similar mode of action to NSC23766. Indeed, further *in vitro* validation of this compound demonstrated the ability of ZINC69391 to interfere with Rac1-Tiam1 binding.^161^ However, it is unclear whether this effect is limited to Tiam1 or extends to other Rac GEFs. Inhibition of Rac1 by ZINC69391 was also associated with reduced epidermal growth factor (EGF)-mediated Rac1 activation and efficient inhibition of cell proliferation, cell cycle progression and cell migration in highly metastatic breast cancer cell lines. More importantly, these anti-metastatic effects were also observed *in vivo*, where ZINC69391 significantly reduced lung colonization in a breast cancer metastasis mouse model. Interestingly, via using ZINC69391 as a lead compound, a more potent analog, 1A-116, was also identified. Similar to ZINC69391, both *in* vitro and *in vivo* assays highlighted the anti-metastatic role of 1A-116. Further analysis of the mode of action of the compound indicated that it exerts its effects through interfering with Rac1-P-Rex1 binding and suppressing Rac1 activation.^161^ Although, it is yet to be determined whether 1A-116 is limited to targeting the Rac1-P-Rex1 binding interface or whether it interferes with other Rac1-GEF complexes, 1A-116 represents a promising Rac1 selective inhibitor that might be of clinical relevance, particularly in cancer. This warrants additional studies to further elucidate its mechanism of action and its clinical applicability.

Given the role of GEFs in dictating Rac1 signaling, compounds that target Rac1 binding to specific GEFs, might, indeed, pave the way towards selective inhibition of Rac1-downstream functions (Fig. 1). Further validation of existing compounds and how they affect the scaffolding function of GEFs is, therefore, important to determine the full therapeutic potential of this mode of Rac1 inhibition.

#### Targeting Rac1-nucleotide interactions

Another important class of inhibitors that target Rac1 activation includes compounds that specifically interfere with Rac1 nucleotide binding, such as EHT 1864. This compound is highly potent relative to NSC23766 with an IC_50_ of 5 μM. It was found to bind with high affinity to Rac1 and its closely related isoforms, Rac1b, Rac2 and Rac3. As a consequence of Rac1 binding, EHT 1864 displaces bound nucleotides resulting in an inert and inactive Rac1 state, which prevents GEF-mediated nucleotide exchange as well as Rac1 binding to downstream effectors (Fig. 2). Importantly, EHT 1864 was shown to effectively block transformation mediated by constitutively active Rac1.^162^ This, together with the fact that it can also bind to the constitutively active splice variant Rac1b, suggest that this type of Rac1 inhibition might prove beneficial for targeting Rac1 activating mutants that have recently been described in a number of cancer types.^80-83^ Inhibition of Rac1 by EHT 1864 has also been shown *in vitro* and *in vivo* to suppress Rac1-driven APP processing and to decrease amyloid-β production.^163^ Thus, this mode of Rac1 inhibition also represents a potential therapeutic avenue in Alzheimer's disease.

#### Additional insights for targeting Rac1 activation

In addition to Rac1-GTP loading by GEFs, Rac1 has also been shown to undergo post-translational modifications that help regulate its GTP-bound state. For example, binding of Rac1 to protein inhibitor of activated STAT3 (PIAS3), a small-ubiquitin related modifier (SUMO) E3-ligase, and its subsequent SUMOylation in response to HGF treatment leads to the maintenance of the GTP-bound state of Rac1, thus stimulating lamellipodia formation as well as cell migration and invasion. To date, Rac1 represents the only small GTPase reported to undergo SUMOylation. Interestingly, depleting PIAS3 inhibits HGF-mediated Rac1 activation. Similarly, it was shown that a Rac1 mutant that could not be SUMOylated displays reduced activity following HGF stimulation. More importantly, expression of this mutant in Rac1 knockout mouse embryonic fibroblasts (MEFs) failed to restore HGF-induced lamellipodia formation, migration and invasion but rescued a proliferation defect. Thus, inhibiting Rac1 SUMOylation might provide a selective approach to destabilize the active form of Rac1 and suppress the associated Rac1-downstream signaling cascades.^26^ This might be particularly relevant in selectively targeting Rac1 pro-migratory properties in a clinical setting. It is, thus, important to further examine the abundance of this modification in disease models and whether Rac1 activating mutants are also subjected to SUMOylation. Additionally, further characterization of the Rac1-PIAS interaction from a structural perspective will be essential to help develop compounds that efficiently interfere with their interaction site.

### Targeting Rac1 spatial regulation

Post-translational modifications also play a crucial role in Rac1 activation via regulating its subcellular localization. Of particular relevance for Rac1 signaling is membrane targeting, via carboxyl-terminal modifications by lipid moieties.^21,24,29,30,36^ Translocation of Rac1 to the plasma membrane is mediated by a series of modifications that are triggered by Rac1 prenylation. Protein prenylation involves the addition of a 15-carbon farnesyl or a 20-carbon lipophilic geranylgeranyl isoprenoid moiety, through the action of farnesyltransferases (FTases) or geranylgeranyltransferases (GGTases), respectively. The specificity of these enzymes is dictated by the sequence within a CAAX motif, where C represents a cysteine residue to which the isoprenoid moiety is transferred, the 2 A residues represent aliphatic amino acids and the X denotes the terminal amino acid. Given that Rac1's canonical CAAX motif ends with a leucine, Rac1 is typically recognized by GGTase type I (GGTase I), which mediates the covalent attachment of a geranylgeranyl isoprenoid moiety to cysteine 189. In turn, this facilitates the cleavage of the AAX amino acids by the RAS-converting CAAX endopeptidase (RCE1) and the methylation of Rac1 on the isoprenylated cysteine residue by the isoprenylcysteine carboxyl methyltransferase (ICMT). Together, this increases the hydrophobicity of Rac1 and mediates its association with the plasma membrane, where it can be activated.^24,30^ Prenylation also primes Rac1 for S-palmitoylation, in which a 16-carbon fatty acid palmitate is covalently attached to cysteine 178. This, in turn, enhances Rac1's stability and membrane association (Fig. 3). Indeed, inhibition of Rac1 palmitoylation disrupts Rac1 localization and suppresses Rac1-driven cell spreading and migration.^21,29^

#### Targeting Rac1 lipid modifications

Given the importance of these modifications in mediating Rac1 localization, activation and downstream signaling, blocking Rac1 lipid modifications represents an additional mode for targeting Rac1 signaling. Three major classes of compounds affecting this regulatory mechanism have been developed and show promising results in hindering disease progression (Fig. 3). The first class, known as statins function by inhibiting the 3-hydroxy-2-methylglutaryl-coenzyme A reductase (HMG-CoA reductase), a rate-limiting enzyme in the isoprenoid pathway. Statins have proved quite successful in primary and secondary prevention of cardiovascular diseases.^164^ Although this was initially attributed to their role in lowering cholesterol, several studies have now identified cholesterol lowering-independent effects. Of particular relevance is the statin-mediated inhibition of isoprenoid synthesis, products of which play a key role in the spatial regulation of small GTPases (Fig. 3A). As a consequence, statins have been shown to improve endothelial dysfunction, attenuate vascular remodeling as well as suppress inflammatory responses associated with cardiovascular diseases. Interestingly, statins suppress Rac1 carboxyl-terminal methylation, a key step required for the proper localization of Rac1.^165^ Consistently, statins have been shown to affect Rac1-mediated cellular effects. For example, simvastatin treatment suppresses Rac1-dependent MMP-1 production and release in SMCs cultured on collagen concomitant with a reduction in GTP-bound Rac1 levels. Additionally, this was associated with reduced collagen degradation.^166^ As indicated earlier, Rac1 contributes to the accumulation of SMCs in the inner layer of arteries in atherosclerosis through regulating SMCs' migration.^104^ Thus, inhibiting Rac1-mediated SMCs' migration represents an important mechanism of action of this class of drugs in cardiovascular diseases. Importantly, co-incubation of simvastatin with mevalonate and geranylgeraniol, precursors of the geranylgeranyl moiety that associates with Rac1, completely abolished the inhibitory effects of simvastatin on Rac1-mediated MMP-1 production, thereby demonstrating that statins do indeed exert their effects through inhibiting protein prenylation.^166^ Despite this evidence, it is still unclear whether the statin-mediated inhibition of protein prenylation observed experimentally is relevant in a clinical setting. Thus, it would be informative to examine the effect of statins on protein prenylation in samples obtained from treated patients.

Intriguingly, recent data also uncovered a prenylation-independent mode of action in which the protective effects of statins were attributed to the degradation of the nuclear pool of Rac1.^167^ Indeed, a number of imaging-based studies have demonstrated the presence of Rac1 in the nucleus,^168,169^ which is mediated via a nuclear localization sequence (NLS) embodied in the polybasic region (PBR) within Rac1.21 Interestingly, the nucleocytoplasmic shuttling of Rac1 has been implicated in regulating cell cycle progression,^170^ nuclear membrane shape and actin polymerization in the nucleus.^171^ Therefore, by stimulating nuclear Rac1 degradation, the protective effects of statins might also be a direct consequence of suppressing nuclear Rac1, although more studies are required to further elucidate the full spectrum of nuclear Rac1-mediated functions, to accurately assess this possibility. The prenylation-independent effects of statins might also be of clinical importance in other diseases, such as cancer. For example, analysis of Rac1 expression in cervical pre-malignant biopsies indicated increased expression of Rac1 in biopsies with low-grade squamous intraepithelial lesions (SIL) and high-grade SIL, compared to biopsies without SIL. Importantly, nuclear Rac1 was only detected in samples with SIL. Consistently, nuclear Rac1 was observed in the cervical cancer cell lines C33A and SiHa, but not in non-tumorigenic cells, such as HaCat, suggesting a role of nuclear Rac1 in disease progression.^172^ It was also shown that Rac1 nuclear accumulation mediates tumor cell invasion due to increased RhoA signaling in the cytoplasm.^171^ Thus, by promoting the degradation of nuclear Rac1, statins might also be beneficial for targeting cancer metastasis. Yet, it would be important to thoroughly examine the anti-metastatic potential of statins in various cancer disease models, to accurately ascertain their therapeutic potential and the underlying molecular mechanisms. It is important to note, however, that statins have also been shown to suppress signaling cascades mediated by other small GTPases, such as RhoA and, thus, their protective effects are, most likely, not solely dependent on Rac1.^164^

The second class of compounds, which interfere with Rac1 spatial regulation, are GGTase I inhibitors (GGTIs). These compounds target GGTase I, thereby inhibiting protein prenylation (Fig. 3B). *In vivo* studies demonstrate the anti-tumorigenic effects of this class of compounds. For example, inhibition of GGTase I in a human pancreatic cancer xenograft mouse model was associated with reduced tumor growth concomitant with inhibition of protein geranylgeranylation.^173^ Similar effects were also observed in a non-small cell lung cancer xenograft model.^174^ Together, these studies highlight the benefit of GGTIs as anti-cancer therapies. Although the full spectrum of GGTIs' protein targets is yet to be elucidated, it is speculated that these compounds will affect multiple small GTPases. Importantly, it has been shown that Rac1 is a target of GGTIs, with GGTI treatment impeding Rac1-driven membrane ruffling.^175^ Interestingly, one GGTI, GGTI-2418, has entered clinical trials.^176^ It would be interesting to examine, whether in a clinical setting this inhibitor suppresses Rac1-mediated pro-tumorigenic effects. Additionally, while Rac1 is typically geranylgeranylated, expression of a farnesylated carboxyl-terminal Rac1 mutant was shown to retain Rac1 signaling abilities, including transformation and membrane ruffling.^175^ Although this has not been reported for geranylgeranylated proteins, it would still be important to investigate whether inhibition of Rac1 geranylgeranylation by GGTIs might lead to an isoprenoid modification switch, a phenomenon observed with K-Ras and neuroblastoma Ras viral (v-ras) oncogene homolog (N-Ras) following treatment with FTase inhibitors.^177^

Inhibitors of palmitoylation constitute the third class of compounds targeting Rac1 spatial regulation (Fig. 3C). Protein palmitoylation has been implicated in a number of human diseases, including cancer, cardiovascular diseases and neurodegenerative disorders. This is mainly attributed to its role in modulating the proper localization and activity of a number of proteins that are critical for the development of human diseases.^178^ This, in turn, sparked the interest for the development of palmitoylation inhibitors. Palmitoylation is mediated by palmitoyl acyltransferases (PATs). As such, a number of inhibitors have been developed against PATs.^178,179^ Although the therapeutic potential of these compounds is yet to be elucidated in different diseases models, inhibition of palmitoylation has been shown to induce an increased perinuclear localization of Rac1 as opposed to its normal subcellular distribution at the cytoplasm, plasma membrane and nucleus. This effect was also associated with decreased Rac1 GTP loading.^29^ This suggests that inhibition of palmitoylation in a clinical setting, while probably, influencing the localization of other proteins that undergo this modification, might also target Rac1 signaling cascades. This warrants additional *in vitro* and *in vivo* studies that examine the effect of PAT inhibitors on Rac1-driven cellular processes.

#### Additional insights for targeting Rac1 spatial regulation

Although the compounds highlighted above target Rac1 in a non-selective manner, they provide insight into the therapeutic benefits of targeting mechanisms that regulate Rac1 localization. RhoGDIs, therefore, represent an interesting class of proteins that can aid in the development of selective Rac1 inhibitors that interfere with Rac1 localization. Functional characterization of RhoGDIs revealed that they inhibit Rac1 signaling via stabilizing the GDP-inactive form through binding to the carboxyl-terminus of GDP-bound Rac1, thus masking the lipid moieties responsible for plasma membrane translocation. As a consequence, GDP-bound Rac1 is sequestered in the cytoplasm where GTP loading cannot occur (Fig. 3).^180,181^ Additionally, determination of the crystal structure of Rac1 in complex with RhoGDI also provided important details regarding their interaction sites and how RhoGDIs mask Rac1 lipid modifications.^182^ Utilizing this information to design structure-based virtual screening approaches might, therefore, facilitate the development of small molecules that mimic the functional role of RhoGDIs, which, in turn, can be used to suppress Rac1 signaling. This strategy might prove particularly useful in combination with GGTIs, especially if Rac1 undergoes an isoprenoid modification switch *in vivo*, since it will provide an efficient method for masking Rac1 lipid modifications despite the nature of the modification. However, the specificity of such compounds will need to be tested against other small GTPases that might share similar interaction sites with RhoGDIs.

### Targeting specific Rac1 downstream effects

Although all of the compounds described above ultimately function through inhibiting Rac1-effector binding indirectly, via reducing Rac1-GTP levels, inhibition of specific Rac1-effector interactions is perhaps a more direct way to block specific Rac1-driven cellular effects whilst not affecting other downstream signaling cascades. While promising, this mode of Rac1 targeting requires detailed knowledge of pathological Rac1 signaling cascades and the identification of Rac1-effector complexes that are more relevant under pathological but not physiological conditions, which is often difficult. However, there are a number of examples that highlight the importance of pursuing this avenue further. For instance, the therapeutic potential of selectively inhibiting Rac1-mediated ROS production has instigated the search for compounds that target Rac1 binding to its downstream effector p67^*phox*^, in an attempt to suppress Rac1-mediated assembly and activation of NOX2. This led to the identification of Phox-I class inhibitors, such as Phox-I1, as novel Rac1 inhibitors (Fig. 4). Indeed, functional analysis of Phox-I1 demonstrated the efficiency of this compound in inhibiting ROS production in neutrophils, via blocking the interaction site of p67^*phox*^ with Rac1.^183^ Although, additional validation of this class of compounds is required, targeting Rac1- p67^*phox*^ binding promises to have protective effects in a number of human diseases. The identification of Phox-I1 also highlights the feasibility of targeting Rac1 binding to specific downstream effectors. Thus, adopting a similar approach to target Rac1-effector protein complexes involved in disease progression will provide a highly selective mode of Rac1 signaling inhibition that help retain its protective and physiological functions and reduce side effects associated with treatment. Although this requires a detailed understanding of protein structures and the binding interface between Rac1 and the respective effectors, advancements in the field of structural biology will soon allow more rapid screening for Rac1-effector targeting.

In addition to targeting Rac1-effector interactions, compounds that inhibit specific Rac1 downstream signaling cascades have also been described. For example, 8-hydroxy-2-deoxyguanosine (8-OHdG), a naturally occurring marker for oxidative stress, has been shown to specifically target Rac1-driven NADPH oxidase activation and ROS production (Fig. 4).^7^ Indeed, Rac1 inhibition following 8-OHdG treatment was associated with protective effects in an *in vivo* atherosclerosis model, with treated animals displaying a significant reduction in vessel lumen occlusion due to reduced ROS production in the arterial wall as well as inhibition of macrophage accumulation.^184^ This further highlights the therapeutic benefit of targeting specific Rac1 downstream effects and warrants the search for more potent inhibitors that selectively inhibit Rac1 signaling cascades that are implicated in human diseases.

## Concluding remarks and future perspectives

Deregulation of Rac1 signaling is implicated in several human diseases. Therefore, targeting Rac1 in a clinical setting presents an exciting therapeutic opportunity. Indeed, as highlighted in this review, several screening approaches have been developed that led to the identification of a number of Rac1 inhibitors. While some of these compounds show promising results, it is evident that more potent inhibitors are yet to be identified. This, together with the lack of extensive *in vivo* validation and clinical evaluation, make it difficult to accurately assess the benefits and potential drawbacks of targeting Rac1. However, advancements in high-throughput screening assays together with our expanding knowledge of the mechanisms involved in regulating Rac1 signaling pave the way toward the development of highly specific and context-dependent Rac1 inhibitors. To facilitate this process further, it would be important to pinpoint regulatory mechanisms that are relevant under physiological and pathological conditions. In particular, understanding the mechanisms involved in dictating specific Rac1 downstream cellular outcomes will aid in the discovery of potent compounds that target specific Rac1 downstream signaling cascades. It is important to note, however, that while targeting Rac1 binding to specific effectors provides a directed approach to inhibiting selective Rac1 downstream cascades, it is equally important to investigate the role of upstream regulators, such as GEFs, in dictating Rac1 signaling. In fact, rational design of additional compounds that selectively interfere with one Rac1-GEF complex and not the other, might provide an alternative and effective approach for the selective inhibition of specific Rac1 downstream effects. This is particularly relevant given the large repertoire of inhibitors identified to date that target Rac1-GEF association, together with the documented role of GEFs in influencing GEF-specific Rac1 downstream signaling cascades. Additionally, as highlighted in this review, there are multiple ways by which Rac1 inhibition can be achieved. Thus, it would also be important to investigate the potential benefits of combining several targeting approaches to accommodate different cellular contexts. For example, combining an inhibitor that targets Rac1-nuceotide binding with a compound that interferes with specific Rac1-GEF complexes, might help effectively inhibit constitutively active Rac1 mutants, via inactivating Rac1 as well as interfering with GEF scaffolding functions. However, regardless of the approach adopted, it will be necessary to supplement the overwhelming *in vitro* evidence, highlighting the benefits of targeting Rac1, with additional validation in different preclinical disease models, in order to truly assess the benefit and drawbacks of inhibiting Rac1.

## Abbreviations

8-OHdG: 8-hydroxy-2-deoxyguanosine
ADAMTS-5: ADAM metallopeptidase with thrombospondin type 1 motif 5
AdoHcy: S-adenosyl- _L_ homocysteine
AdoMet: S-adenosyl methionine
ALS: Amyotrophic lateral sclerosis
ANF: Atrial natriuretic factor
APC: Adenomatous polyposis coli
APP: Amyloid precursor protein
Bfgf: Basic fibroblast growth factor
BRAF: B-Raf proto-oncogene, serine/threonine kinase
C: Cysteine
Cdc42: Cell division control protein 42 homolog
ClearCa-28: Renal cell carcinoma
COLX: Type X collagen
DH: Dbl homology
ECM: Extracellular matrix
EGF: Epidermal growth factor
EMT: Epithelial-to-mesenchymal transition
ER: Endoplasmic reticulum
FLII: Protein flightless-1 homolog
FPP: Farnesyl diphosphate
FTase: Farnesyltransferases
GAP: GTPase activating protein
GDI: Guanine nucleotide dissociation inhibitor
GDP: Guanosine diphosphate
GEF: Guanine nucleotide exchange factor
GGPP: Geranylgeranyl diphosphate
GGTase: Geranylgeranyltransferases
GGTase I: GGTase type I
GGTIs: GGTase I inhibitors
GPP: Geranyl diphosphate
GTP: Guanosine triphosphate
GTPase: Guanosine triphosphate phosphohydrolase
H: Hydrogen
H-Ras: Harvey rat sarcoma viral oncogene
HGF: Hepatocyte growth factor
HIV: Human immunodeficiency virus
HMG-CoA reductase: 3-hydroxy-2-methylglutaryl-coenzyme A reductase
I: Isoleucine
IB2/JIP2: Mitogen-activated protein kinase 8 interacting protein 2
ICMT: Isoprenylcysteine carboxyl methyltransferase
IPP: Isopentenyl diphosphate
IRSp53: Insulin receptor tyrosine kinase substrate p53
JNK: Jun N-terminal kinase
K-Ras: Kirsten rat sarcoma viral oncogene
LPS: Lipopolysaccharide
LRRK2: Leucine-rich repeat kinase 2
MAPK: Mitogen-activated protein kinases
MDCKII: Madin-Darby canine kidney II
MEFs: Mouse embryonic fibroblasts
Min: Multiple intestinal neoplasia
MKK3: Mitogen-activated protein kinase kinase 3
MLC: Myosin light chain
MLK3: Mixed lineage kinase 3
MMP: Matrix metalloproteinase
Mtss1: Metastasis suppressor-1
N: Asparagine
N-Ras: Neuroblastoma Ras viral (v-ras) oncogene homolog
NADPH: Nicotinamide adenine dinucleotide phosphate
NOX2: NADPH oxidase 2
O_2_^.−^: Superoxide anion
OCRL1: Inositol polyphosphate 5-phosphatase OCRL-1
P: Proline
P-Rex1: Phosphatidylinositol-3, 4, 5-trisphosphate-dependent Rac exchange factor 1
p38: Mitogen-activated protein kinase 14
PAK: p21 activated kinase
PAT: Palmitoyl acyltransferase
PBR: Polybasic region
PH: Pleckstrin homology
PIAS3: Protein inhibitor of activated STAT3
PIX: PAK-interacting exchange factor
Rac1: Ras-related C3 botulinum toxin substrate 1
Ras-GRF1: Ras protein-specific guanine nucleotide-releasing factor 1
RCE1: RAS-converting CAAX endopeptidase
RhoA: Ras homolog gene family member A
RhoGDI: Rho Guanine nucleotide dissociation inhibitor
ROS: Reactive oxygen species
Runx-2: Runt-related transcription factor 2
S: Serine
SIL: Squamous intraepithelial lesions
SH3: Src-homology 3
SMCs: Smooth muscle cells
SOD1: Cu, Zn superoxide dismutase 1
SUMO: Small-ubiquitin related modifier
Tiam1: T-cell lymphoma invasion and metastasis-1
TIMP: Tissue inhibitor of metalloproteinase
TNF-α: Tumor necrosis factor-α
Trio: Triple functional domain protein
TrioN: Trio N-terminal RhoGEF domain
VE: Vascular Endothelial
VEGF: Vascular endothelial growth factor
WAVE: WASP-family verprolin-homologous protein 2
Y: Tyrosine
